# Study on the Catalytic Performance of Nickel(II) Complexes with Distinct Triazine Support Structures in Ethylene Oligomerization via Different Experiment Designs

**DOI:** 10.3390/molecules30091977

**Published:** 2025-04-29

**Authors:** Xiaobing Wei, Jiahui Li, Dan Li, Lijun Guo, Yanling Xiao, Cuiqin Li

**Affiliations:** 1 College of Economic and Management, Northeast Petroleum University, Daqing 163318, China; dqweixiaobing@126.com; 2 Chemical No.1 Plant, Zibo Qixiang Tengda Chemical Co., Ltd., Zibo 255400, China; hum15045160883@163.com; 3 Provincial Key Laboratory of Polyolefin New Materials, College of Chemistry and Chemical Engineering, Northeast Petroleum University, Daqing 163318, China; lidan201901@163.com (D.L.); guolijun1026@163.com (L.G.)

**Keywords:** covalent organic frameworks, Schiff-base imine, supported catalyst, ethylene oligomerization

## Abstract

Covalent organic frameworks hold great promise for heterogeneous catalysis because of their porous structure for gas adsorption and tunable functionality. Two triazine support materials (MA*m*PA-COF and MA*o*PA-COF) were prepared by using melamine as the linked monomer and meta-phthalaldehyde (MPA) or ortho-phthalaldehyde (OPA) as the sub-construction monomer. Two nickel(II) complexes (Ni@MA*m*PA-COF and Ni@MA*o*PA-COF) based on the synthesized COFs were prepared to use for ethylene oligomerization. The nickel(II) complexes had good catalytic activities in ethylene oligomerization. Moreover, the substituent position of the aldehyde group in the sub-construction monomer had a certain influence on the specific surface area, morphology and catalytic activity. The morphology of Ni@MA*m*PA-COF was spherical, while Ni@MA*o*PA-COF exhibited layered stacking shapes and had a large specific surface area. Ni@MA*o*PA-COF has a higher catalytic activity and higher selectivity for low-carbon olefins in ethylene oligomerization due to its larger specific surface area and smaller pore width. Ni@MA*o*PA-COF has good recyclability and still had excellent catalytic activity after three cycles. Based on the gray correlation analysis and single factor experiment, the reaction pressure was the most important factor affecting the activity of Ni@MA*o*PA-COF in ethylene oligomerization, and the molar ratio of Al/Ni was the main important factor affecting the selectivity.

## 1. Introduction

Ethylene oligomerization, one of the most crucial reactions in petrochemical industry, allows the low-cost conversion of ethylene monomer as the only reaction of raw materials to C_4_-C_24_ high value-added linear alpha olefins (LAOs). These olefins can be used as key intermediates to obtain low-density linear polyethylene (LLDPE), liquid transport fuels, surfactants, and detergents [1,2]. Developments in the petrochemical industry in recent years have led to a sharp increase in demand for LAOs, especially for low-carbon C_4_, C_6_, C_8_ and C_10_ olefins [3]. In view of this, it is extremely important to investigate catalytic systems that can not only effectively promote ethylene oligomerization, but also selectively produce these high value-added intermediates.

The use of heterogeneous catalyst has been extensively investigated and offers the advantages such as the ability to control product selectivity, the increase in the material strength and the reuse from the reaction. In recent years, numerous studies have reported on heterogeneous catalytic systems for the application of ethylene oligomerization, mainly the exploration of catalyst supports, including SiO_2_ [4], molecular sieves [5,6], carbon nanotubes [7,8], ionic liquid [9], porous clay [3], Fe_3_O_4_ nanoparticles [10] and MOFs [11,12], etc. These studies showed that the heterogeneous catalysts have excellent mechanical properties, thermal stability and reusability, which can greatly reduce the operating cost of the ethylene oligomerization process to a certain extent. However, the above catalysts have some drawbacks that limit their application in ethylene oligomerization, such as bad structural controllability, poor thermal stability, difficulty in modification and lack of metal active centers. In this context, developing new heterogeneous catalytic systems is of great significance in the field of ethylene oligomerization.

Covalent organic frameworks (COFs) are novel crystalline organic porous polymers with pre-designed pore structures formed by molecular building blocks with reversible covalent bonds [13]. Compared with other organic porous materials, COFs have the advantages of a high specific surface area, low density, high crystallinity, and adjustable structures of building units. They show promising potential applications in the fields of gas adsorption [14], energy storage [15], photoelectric chemistry [16,17], sensing [18] and heterogeneous catalysis [19,20]. Among various COFs with different structures, Schiff-base COFs are widely used in heterogeneous catalysis for their structure containing the C=N group with strong coordination ability, which can cooperate with different metals to form materials with different catalytic performances. Fan and his co-workers prepared Pd@COF-Ph and Pd@COF-BPh catalysts with different pore diameters, and the catalysts exhibited superior activity and excellent stability (reused 10 times) for the reduction of 4-nitrophenol, which was attributed to the high utilization of Pd-active sites and the confinement effect of triazinyl-COFs [21]. The imine-linked COF-LZU1, in combination with Pa, has been employed in the Suzuki–Miyaura cross-coupling reaction with excellent catalytic performance (96–98% yields) [22]. COFs can perform in situ polymerization of monomers in channels to produce linear polymers because they provide a safe way to integrate polymer chains into 1D channels. Rozhko and his co-workers first reported novel nickel catalysts with covalent organic frameworks as supports, and the catalysts exhibited excellent catalytic activity and reusability for ethylene oligomerization, with more than 85–90% yields for a narrow scope of C4-C6 olefins [23]. In our previous work, two triazine supports (MAPA-COF and MABD-COF) based on melamine were synthesized in dimethyl sulfoxide (DMSO), as well as the corresponding nickel catalysts (Ni@MAPA-COF and Ni@MABD-COF) for ethylene oligomerization. The results demonstrated that the abovementioned nickel catalysts had good catalytic performance in ethylene oligomerization, and the catalyst with larger specific surface area had higher catalytic activity [24]. COFs obtained in highly polar solvent have low crystallinity because of their excessively fast rate of product precipitation, and the catalysts loaded with nickel had low crystallinity and small pore width.

In order to improve the abovementioned phenomena and develop COFs-based catalysts with higher activity, it is crucial to modify the structure of ethylene oligomerization catalysts, especially the design of their support structure. This affects the relationship between catalyst structure and catalytic performance. Therefore, based on our previous research on the possible application of COFs in ethylene oligomerization, this work attempts to further improve the microstructure of COFs ligands with meta-phthalaldehyde (MPA) or ortho-phthalaldehyde (OPA) as sub-construction monomers in the mixed solvent to obtain COFs-based catalysts with high activity. The structure–activity relationship between the structure of the nickel(II) complexes-based triazine support materials and the catalytic behavior of ethylene oligomerization will be explored.

## 2. Results and Discussion

### 2.1. Synthesis and Characterization

**Scheme 1:**
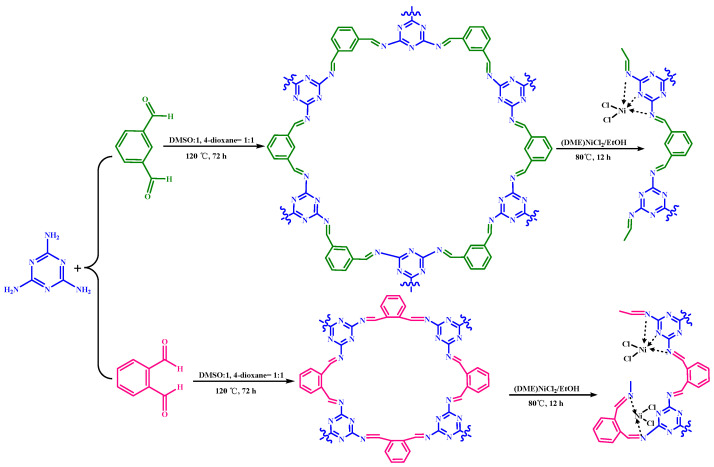
Synthetic process of triazine support materials and the corresponding supported nickel(II) catalysts.

Two kinds of triazine COFs (MA*m*PA-COF and MA*o*PA-COF) were prepared using the Schiff-base reaction between melamine and aromatic dialdehyde (MPA and OPA) (Scheme 1) with HAc (6 mol/L) as the catalyst [25]. In order to obtain the COFs with high crystallinity and high specific surface area, the mixed solvent of DMSO and 1,4-dioxane was used in the synthesis process. The corresponding catalysts (Ni@MA*m*PA-COF and Ni@MA*o*PA-COF) were produced with (DME)NiCl_2_ as the nickel precursor (Scheme 1). In order to confirm the successful synthesis and the difference in the triazine support materials and their nickel(II) complexes, the chemical constitution and morphology of the prepared COFs were characterized by FT-IR, SEM, XRD, BET and TG, and the nickel(II) catalysts were verified by FT-IR, ICP, SEM, EDX, XRD, BET, XPS and TG.

**Figure 1:**
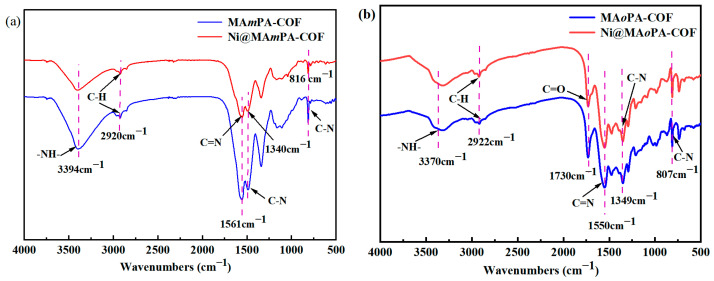
FT-IR spectra of (**a**) MA*m*PA-COF and Ni@MA*m*PA-COF, (**b**) MA*o*PA-COF and Ni@MA*o*PA-COF.

As shown in Figure 1a, the peaks at 3394 cm^−1^, 2920 cm^−1^, 1340 cm^−1^ and 816 cm^−1^ could be attributed to the stretching vibrations of the -NH- bond, C-H bond and C-N bond of MA*m*PA-COF and Ni@MA*m*PA-COF, respectively. The stretching peaks at 1561 cm^−1^ for MA*m*PA-COF and Ni@MA*m*PA-COF were observed, which indicated that the melamine and MPA have reacted to formed imine linkages. In addition, the stretching vibrations of C=O which should be present in the spectra of support and pre-catalyst disappeared, further denoting that the MPA monomer has been converted to a C=N bond. As shown in Figure 1b, the wide absorption peak at 3370 cm^−1^ was in accordance with the stretching pattern of -NH- groups in the aromatic ring. The distinct bands at 1550 cm^−1^ also corresponded to the stretching vibration of the imine bond (-C=N) and indicated the successful formation of Schiff-base COF. The peaks centered at 1349 and 807 cm^−1^ were attributed to the aromatic stretching vibrations of the C-N band. A typical band of aldehyde group appeared at 1726 cm^−1^ of the spectra, indicating the presence of unreacted aldehyde in the samples, which further confirmed that MA*m*PA-COF and MA*o*PA-COF had different architectural models [26]. For Ni@MA*m*PA-COF and Ni@MA*o*PA-COF, there were small changes between the spectra of the supports before and after the coordination with (DME)NiCl_2_.

**Figure 2:**
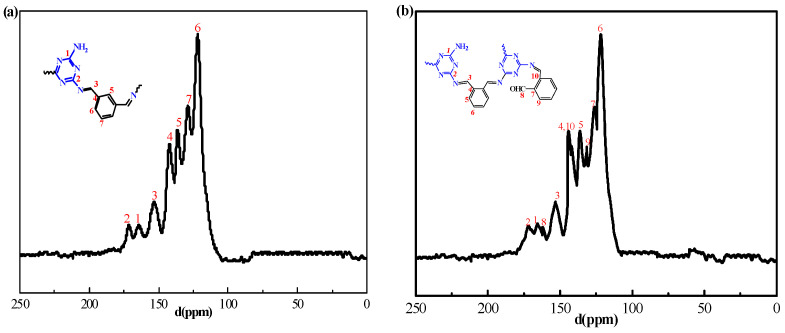
^13^C NMR spectra of (**a**) MA*m*PA-COF and (**b**) MA*o*PA-COF.

The formation of the imine bond (C=N) was confirmed from the 13C solid-state nuclear magnetic resonance (NMR) spectra of MA*m*PA-COF and MA*o*PA-COF. The ^13^C NMR spectrum showed the characteristic resonance of the imine carbon (C=N) peak of MA*m*PA-COF at about 153.1 ppm (Figure 2a). The peak at about 164.6 ppm could reflect the existence of the carbon atoms connected with the primary amine group in the framework, which was consistent with the results of FTIR. The ^13^C NMR spectrum showed the characteristic resonance of the imine carbon (C=N) peak of MA*o*PA-COF at about 152.8 ppm (Figure 2b). The peak at about 165.6 ppm could reflect the existence of the carbon atoms connected with the primary amine group (-NH_2_) in the framework, and the peak at about 161.7 ppm was attributed to the carbon atoms of terminal aldehyde moiety (-CHO), which was consistent with the results of FTIR.

**Figure 3:**
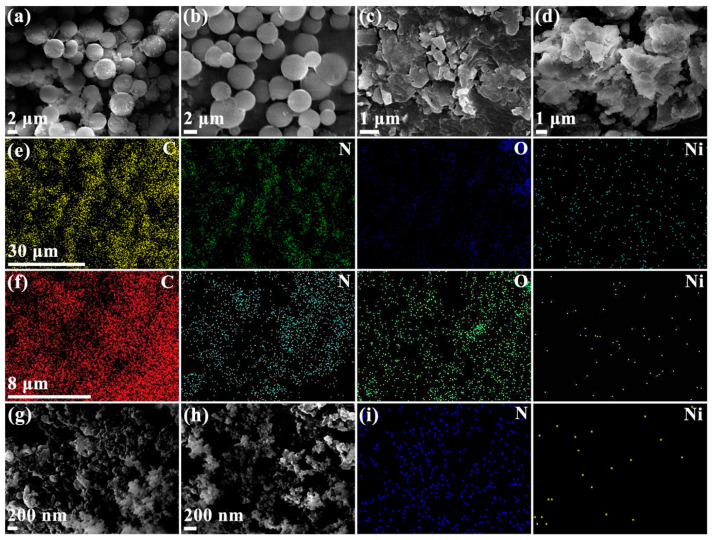
SEM images of MA*m*PA-COF (**a**), Ni@MA*m*PA-COF (**b**), MA*o*PA-COF (**c**), Ni@MA*o*PA-COF (**d**), MAPA-COF (**g**) [24], Ni@MAPA-COF (**h**) [24] and energy-dispersive X-ray spectroscopy (EDX) of Ni@MA*m*PA-COF (**e**), Ni@MA*o*PA-COF (**f**) and Ni@MAPA-COF (**i**) [24].

In order to further confirm the difference in the architectural models of MA*m*PA-COF and MA*o*PA-COF and their corresponding nickel complexes, their morphology and elemental distribution were investigated by SEM and EDX. The melamine and MPA monomers had interacted to generate spherical particles with a rough surface and layered particles with a small size (Figure 3a). Their morphologies were different from the morphology of MAPA-COF (Figure 3g) synthesized with melamine and *p*-phthalaldehyde as materials in our previous work [24]. It can be seen from the image in Figure 2b that the spherical particles with smooth surfaces were observed and the layered particles decreased after MA*m*PA-COF complexation with nickel. Interestingly, the irregular masses were found with melamine and OPA as materials (Figure 3c). This was completely different from MA*m*PA-COF. The morphology of Ni@MA*o*PA-COF (Figure 3d) presented irregular lamellar accumulation after supporting nickel, which was different with the morphology of Ni@MA*m*PA-COF (Figure 3b) and Ni@MAPA-COF (Figure 3h). The results indicated that the chemical structure of the sub-constructing monomer affected the morphology of the synthesized COFs. The substituents on the benzene ring of the sub-constructing unit are located in ortho-position and close to each other, and the adjacent aldehyde group reacted more thoroughly with the amino group in the melamine via the imine bond [27].

Energy-dispersive X-ray spectra images (Figure 3e,f) show a uniform distribution of C, N and Ni in Ni@MA*m*PA-COF and Ni@MA*o*PA-COF. This show that the nickel moiety was incorporated throughout the structure of the two nickel(II) catalysts. Table 1 also summarizes the elemental composition and nickel content of each nickel catalyst, measured by EDX and ICP-AES. Compared with the Ni@MAPA-COF (Figure 3i) synthesized in our previous work [24], the content of nickel in Ni@MA*m*PA-COF and Ni@MA*o*PA-COF measured by ICP-AES was lower. This indicated that the substituent position of the sub-construction monomer directly affects the coordination ability of the imine bonds to nickel(II). The lowest nickel content in Ni@MA*o*PA-COF was hypothesized to be due to fewer imine sites complexed with nickel in its adjacent COF layers than the other two nickel(II) catalysts. (Scheme 1) [28]. Moreover, since the EDX spectrometer only identifies the nickel element on the surface of the material, the nickel contents measured by EDX is considerably less than that measured by ICP. This indicates that some nickel element was trapped in the pores of the synthesis nickel(II) catalyst. In addition, The O atoms in the Ni@MA*m*PA-COF and Ni@MA*o*PA-COF may be due to the unremoved ethylene glycol dimethyl ether in (DME)NiCl_2_. The measured results of SEM, EDX and ICP confirm that the substituent position of the sub-construction unit had a significant effect on the morphology, coordination mode and nickel content of the synthesized triazine support materials and their corresponding nickel catalysts.

**Figure 4:**
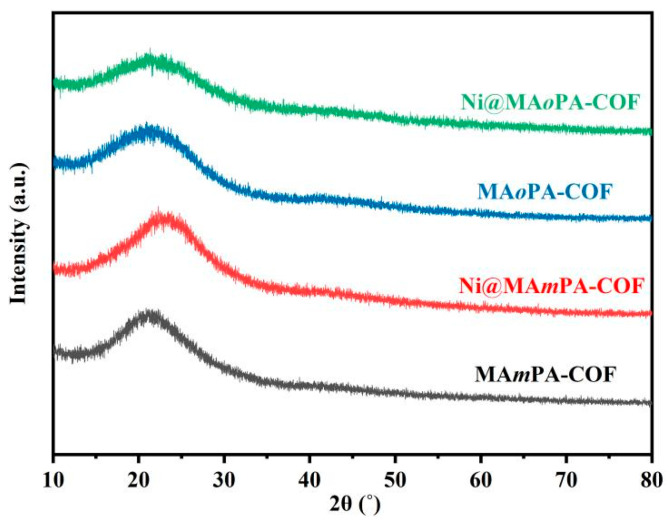
XRD patterns of MA*m*PA-COF, Ni@MA*m*PA-COF, MA*o*PA-COF and Ni@MA*o*PA-COF.

The MAPA-COF synthesized with melamine and p-phthalaldehyde as building blocks in a DMSO solvent in our previous study had low crystallinity [24]. In order to confirm the crystallinity of MA*m*PA-COF and MA*o*PA-COF with DMSO and 1,4-dioxane as solvents in this work, they were tested using XRD. The X-ray diffractograms of MA*m*PA-COF and MA*o*PA-COF and the two nickel catalysts all exhibited distinct broad tympanic peaks around 2θ = 22° and had low crystallinity (Figure 4). The XRD pattern shows that the molecules of these COFs based on the Schiff-base reaction have amorphous arrangement [26]. However, Ni@MAPA-COF had better crystallinity [24]. The difference in crystallinity was because the MAPA-COF molecule has a symmetry-containing linear building monomer (terephthalaldehyde), which facilitates the formation of irregular aggregates with a submicron-sized sheet layer. This caused π-π stacking in the internal layers. Moreover, the high coordination capacity of MAPA-COF leads to increased ordering of Ni@MAPA-COF. The XRD results further indicate that the substituent position of the sub-construction unit had a certain effect on the coordination mode and the crystallinity, which was observed in the SEM and EDX results.

**Figure 5:**
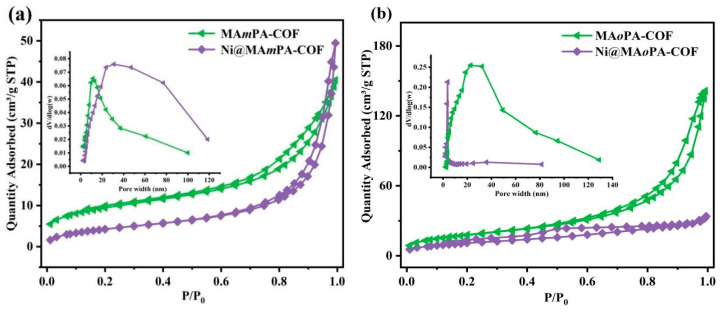
N_2_ adsorption and desorption isotherms and pore size distribution for MA*m*PA-COF, Ni@MA*m*PA-COF (**a**) and MA*o*PA-COF, Ni@MA*o*PA-COF (**b**).

The specific surface areas and porous structures of the synthetic triazine support materials and their corresponding nickel catalysts were obtained using the nitrogen adsorption isotherms at 77 K (Figure 5). As shown in Figure 5a, MA*m*PA-COF and Ni@MA*m*PA-COF exhibited typical type IV isotherms with an extremely small hysteresis loop at low relative pressure (~0.45 P/P_0_) and a sharp increase above P/P_0_ = 0.9. This indicated the presence of mesopores or even macropores, which were presumably caused by the voids in the loose stacking sphere material [23]. The textural properties of COFs and COFs-based catalysts, including Brunauer–Emmett–Teller (BET) surface areas, pore width and total pore volumes of supports and catalysts, are summarized in Table 2. The changes in pore diameters between MA*m*PA-COF and Ni@MA*m*PA-COF confirmed that the entry of nickel into MA*m*PA-COF affected the pore width, along with the shrinkage of 50.3% in the specific surface areas. It was speculated that the increase in pore size was due to the destruction of the microstructure on the surface of the ligand after loading with nickel(II) metal, which leads to the disappearance of some small microspores. The morphology of Ni@MA*m*PA-COF became spherical particles which were smoother than that of the triazine support, and only the large pores were left behind. The synthetic COFs and the corresponding catalyst with OPA as the sub-constituent monomer also showed a typical type IV isothermal profile (Figure 5b), and the specific surface area and pore volume of the catalysts were reduced after loading with metallic nickel (Table 2). After coordination, 37.9% of the surface area of Ni@MA*o*PA-COF was lost, which was much lower than Ni@MA*m*PA-COF. However, the specific surface area, pore width and pore volume of the nickel(II) catalyst with PPA as a sub-constituent unit was much higher than those of the two samples reported here (MA*m*PA-COF and Ni@MA*m*PA-COF). In addition, Ni incorporation has the opposite effect on pore width for MAmPA-COF and MAoPA-COF. The pore width of Ni@MAmPA-COF was higher than that of MAmPA-COF. However, the pore width of Ni@MAoPA-COF was lower than that of MAoPA-COF. The possible reason for this is that Ni@MAmPA-COF has only one tridentate coordination mode (Scheme 1) because of the chemical structure of m-phthalaldehyde. However, Ni@MAoPA-COF may have both coordination modes of bidentate and tridentate coordination (Scheme 1). The competition of these two coordination modes led to changes in the topological structure, resulting in a smaller pore size. This indicates that the specific surface area of nickel(II) catalysts was highly dependent on the unit structure of the materials. The relative positions of the two aldehyde groups reacting with the amino group severely affected the morphology of the samples, which led to the differences in the specific surface area.

**Figure 6:**
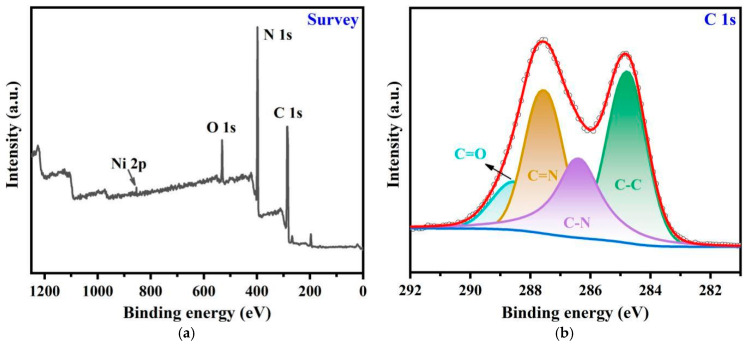

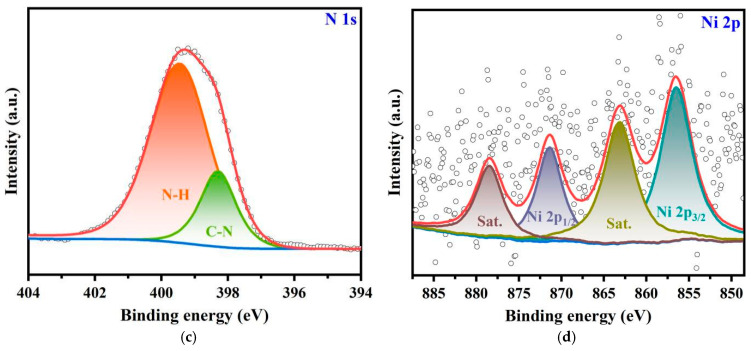
Wide XPS scanning spectrum of Ni@MA*m*PA-COF (**a**) and high-resolution XPS spectra of C 1s (**b**), N 1s (**c**) and Ni 2p (**d**).

The surface composition and the bond valence of Ni@MA*m*PA-COF were investigated by XPS spectroscopy, and the results are shown in Figure 6. The C 1s (287.3 eV), N 1s (399.1 eV), O 1s (533.4 eV) and Ni 2p (855.0 eV) compositions could be observed from the wide XPS scanning spectrum of Ni@MA*m*PA-COF. The characteristic peaks of C1s in the high-resolution XPS spectra were designated as C-C (284.6 eV), C-N (285.7 eV), C=N (287.3 eV) and C=O (288.2 eV). The peaks with binding energies of 398.8 eV and 400.0 eV were attributed to N=C and N-H, respectively. The low peak intensity of Ni 2p was identified in the wide XPS scanning spectrum of Ni@MA*m*PA-COF. To reveal the chemical state of nickel in the complex, the high-resolution Ni 2p XPS spectrum of Ni@MA*m*PA was analyzed, as shown in Figure 6. The Ni 2p spectra displayed two peaks at 856.5 eV and 871.5 eV, corresponding to Ni 2p_3/2_ and Ni 2p_1/2_, respectively. And the spectrum of Ni 2p showed that there were two distinct satellite peaks at 861.53 eV and 879.52 eV [29]. The low content of Ni^2+^ ions might be caused by the weaker coordination effect of N atoms with (DME)NiCl_2_. Due to the six-membered triazine pyridine ring synthesized with MPA as a sub-constituent unit being too large, the Ni^2+^ ions coordinated to a small fraction of N atoms, forming a weak complex, which results in a low peak intensity of Ni2p [23]. It could be seen from the ICP results that the nickel content of Ni@MA*m*PA-COF is 5.76%, and low metal content was difficult to detect using a photoelectron spectrometer.

**Figure 7:**
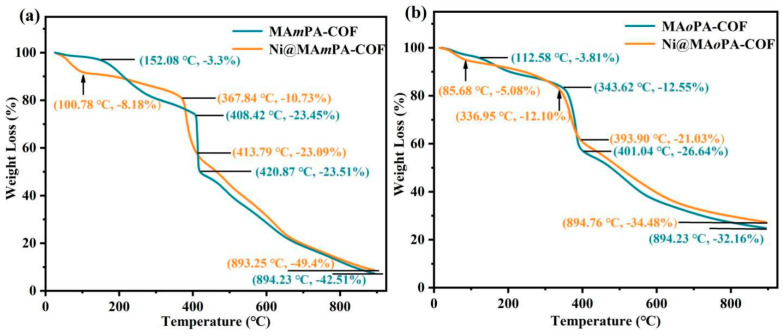
TGA curves of MA*m*PA-COF, Ni@MA*m*PA-COF (**a**) and MA*o*PA-COF, Ni@MA*o*PA-COF (**b**).

Dried samples were subjected to thermogravimetric analysis (TGA) under nitrogen to research the interactions between the supports with (DME)NiCl_2_ (Figure 7). According to the thermograms, the weight loss of MA*m*PA-COF and MA*o*PA-COF could be explained by the loss of water and gas at temperatures of 152.08 °C and 112.58 °C, respectively. MA*m*PA-COF and MA*o*PA-COF showed weight loss of 23.45% and 12.55% with increasing temperature in the ranges of 152.08–408.42 °C and 112.58–343.62 °C, respectively [30]. In the third stage, a rapid mass loss (approximately 23.51% and 26.64%) of MA*m*PA-COF and MA*o*PA-COF caused by the thermal decomposition of the primary amine group (-NH_2_) in the framework was noticed. The weight loss of MA*o*PA-COF was higher than that of MA*m*PA-COF; this was because there was the unreacted aldehyde groups (-CHO) in the framework of MA*m*PA-COF, and its thermal decomposition leading to an increase in weight loss [31]. The mass loss of MA*m*PA-COF and MA*o*PA-COF approached 42.51% and 32.16%, respectively, when the temperature was elevated to 894.23 °C. This was generated by the degradation of the COFs structure, which led to the decomposition of the carbon skeleton. Ni@MA*m*PA-COF and Ni@MA*o*PA-COF displayed a negligible weight loss (about 8.18% and 5.08%) at initial temperature (100.78 °C and 85.68 °C), which could be ascribed to the volatilization of water. The initial dissociation of (DME)NiCl_2_ from MA*m*PA-COF and MA*o*PA-COF prompted the second weight loss stage for the two COFs-based catalysts in the temperature range of 100.78~367.84 °C and 85.68~336.95 °C, respectively [24]. The collapse of the entire support backbone, which culminated in the complete dissociation of the carbon chains, was responsible for the mass loss in the last stage of the nickel(II) catalysts [32]. Moreover, the thermal stability of the nickel(II) catalysts decreased compared to the unloaded support material, which could be explained by the weak coordinated covalent bond between nickel ions and triazine COFs.

### 2.2. Ethylene Oligomerization Catalyzed by Ni@MAmPA-COF

The above-characterized results show that the substituent positions of the sub-construction unit had a significant effect on the morphology, coordination mode and nickel content of the synthesized supports (MAmPA-COF and MAoPA-COF) and their corresponding nickel catalysts (Ni@MAmPA-COF and Ni@MAoPA-COF). In order to investigate the effect of the substituent positions of the sub-construction unit on the catalytic property, the optimal oligomerization conditions were studied and screened using Ni@MA*m*PA-COF with a higher nickel content as a precatalyst.

#### 2.2.1. Influence of Reaction Parameters on the Catalytic Properties of Ni@MAmPA-COF

**Figure 8:**
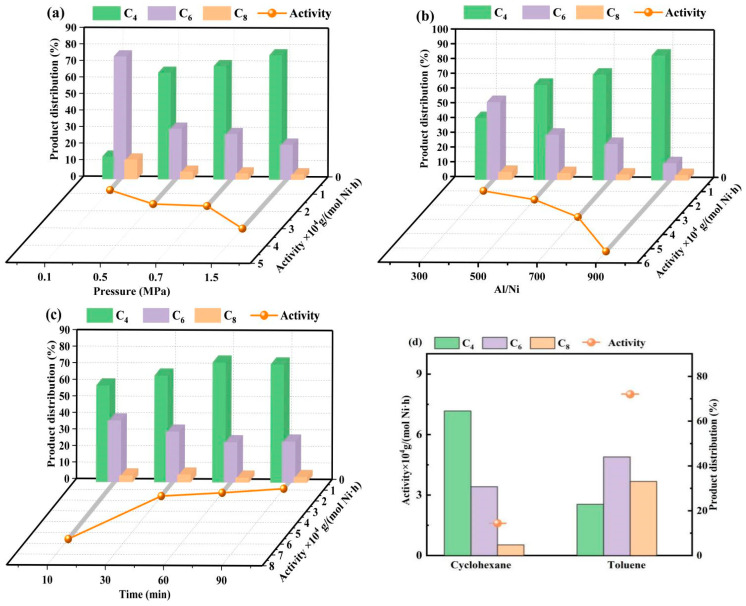
Effects of reaction parameters on the catalytic properties of Ni@MA*m*PA-COF: effects of (**a**) ethylene pressure, (**b**) Al/Ni ratio, (**c**) reaction time and (**d**) reaction solvent.

Ethylene pressure plays an important role in ethylene oligomerization, because the elevated pressure contributes to the high solubility of ethylene in the solvent. More ethylene was available for active sites to improve the catalytic activity [33]. The catalytic activity of Ni@MA*m*PA-COF reached a maximum value of 3.0 × 10^4^ g/(mol Ni·h) at 1.5 MPa (Figure 8a). Interestingly, the catalytic activity of Ni@MA*m*PA-COF increased from 1.6 × 10^4^ g/(mol Ni·h) to 1.7 × 10^4^ g/(mol Ni·h) when the ethylene pressure increased from 0.5 MPa to 0.7 MPa. The results indicated that the pressure had little effect on the catalytic activity of Ni@MA*m*PA-COF. Increased reaction pressure was due to an increase in the selectivity of butane in oligomers, as demonstrated by the increase in the amount of C_4_ from 54.67% to 75.32% (Figure 8a). This was because the high pressure contributed more to *β*-H elimination than the chain propagation in the oligomerization process, and the selectivity (30.72%) to C_6_ maximized at 0.5 MPa. Comprehensively considering the influence of ethylene pressure on catalytic activity and selectivity toward C_4_ and C_6_, the ethylene pressure of 0.5 MPa was determined as the optimum value for further reaction. The catalytic activity of Ni@MA*m*PA-COF reached the optimum activity of 5.2 × 10^4^ g/(mol Ni·h) at the Al/Ni ratio of 900, which suggests that ethylene oligomerization reaction demands sufficient MAO to activate more active sites (Figure 8b). Variation in Al/Ni was also found to affect the product distribution of Ni@MA*m*PA-COF, as evidenced by an evidently higher selectivity for C_4_ and a relatively low selectivity for C_6_ (the value of Al/Ni was from 300 to 900), which implied that higher amount of MAO might contribute to the reaction of *β*-H elimination. Combined with the effect of the Al/Ni ratio on catalytic activity and selectivity toward C_4_ and C_6_, the optimum Al/Ni ratio for the subsequent reaction was determined to be 500.

The lifetime of Ni@MA*m*PA-COF was investigated, with the reaction time varied from 10 to 90 min, and the results are revealed in Figure 8c. As the reaction time prolonged from 10 to 90 min, the catalytic activity continued to decline. The decrease in catalytic activity could be explained by the oligomer continuously interfering with the contact between the active center and ethylene monomer. Furthermore, the reaction time of Ni@MA*m*PA-COF also had a slight influence on the product distribution. An increase in reaction time resulted in a higher percentage of butene, combined with a slight decrease in selectivity toward C_6_ and C_8+_ during the 10 to 60 min. Moreover, the percentage of C_4_-C_6_ olefin in the oligomers was above 95.26%; in particular, the content of C_4_ and C_6_ was relatively higher and up to 64.54% and 30.72% at 30 min, respectively. The dispersibility of the heterogeneous catalysts and the solubility of ethylene in solvent have a significant impact on the catalytic property of the heterogeneous catalyst. The catalytic performance of Ni@MA*m*PA-COF was investigated in cyclohexane and toluene (Figure 8d). Cyclohexane was more suitable for ethylene oligomerization compared with toluene as the solvent, although higher catalytic activity of Ni@MA*m*PA-COF was recorded in the toluene, which could be attributable to the good polarity of toluene [34]. Moreover, the selectivity toward C_4_-C_6_ was higher in the solvent of cyclohexane than that of toluene; therefore, cyclohexane would be the preferential solvent.

To quickly find the characteristic variables that are most relevant to catalytic activity and selectivity for low-carbon olefins, the two target variables of the catalytic activity and the selectivity of low-carbon olefins were selected and the gray relational degree was calculated using Gray System Theory Modeling Software, version 7.0 (Table 3). The reaction pressure was the main factor affecting the catalytic activity. The higher pressure in the reaction system led to the higher solubility of ethylene, which was favorable for the injection of ethylene into the nickel-active center, and the catalytic activity increased. However, the molar ratio of Al/Ni was the main factor affecting the low-carbon selectivity. More cocatalysts were used to fully activate the Ni@MA*m*PA-COF to a more active center to enhance catalytic activity, while further increasing the molar ratio of Al/Ni may lead to increase in the rate of chain transfer to aluminum to form low-carbon olefins.

#### 2.2.2. Influence of Chemical Structure on the Catalytic Properties of Catalysts

**Figure 9:**
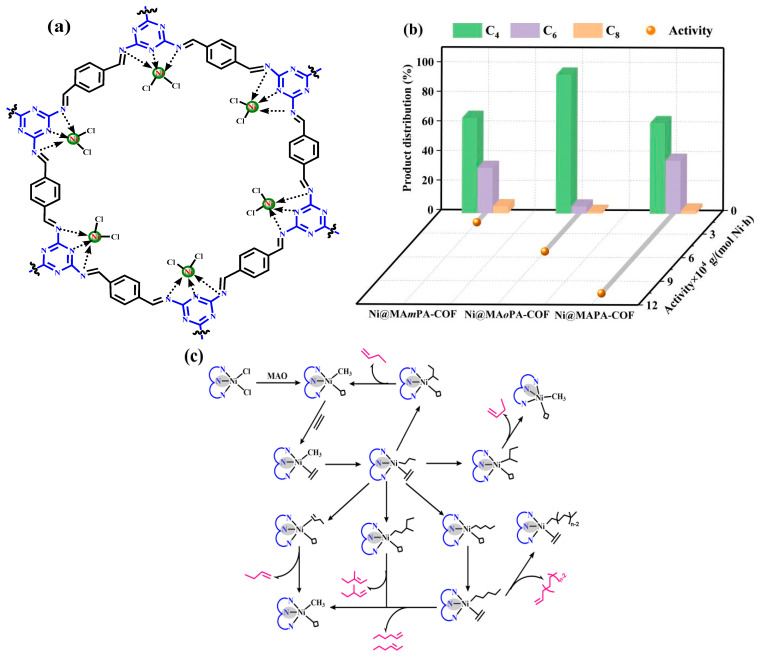
Catalytic performance of nickel catalyst-based triazine COFsL: (**a**) chemical structure; (**b**) catalytic properties; (**c**) possible mechanism of ethylene oligomerization. Reaction conditions: 2 µmol precatalyst, MAO (Al/Ni = 500), 20 mL cyclohexane, 25 °C, 0.5 MPa, 30 min.

It is well known that the possible mechanism of ethylene oligomerization for the nickel(II) catalyst is as shown in Figure 9c. The active sites with empty orbitals were converted upon activation by MAO. The incorporated ethylene molecules coordinated with the active sites to form metal–alkyl groups, and chain extension occurred. At the same time, the insertion of the ethylene molecule is followed by a β-H elimination reaction, leading to the generation of short-chain oligomers and the restoration of the active site [35]. For the heterogeneous catalysts, the nature of the supports, the coordination mode and the dispersion and content of the metal active center in the supports have an important effect on their catalytic performance. In view of the above results on Ni@MA*m*PA-COF in ethylene oligomerization, the effect of the chemical structures of the synthesized triazine supports and the nickel content on the catalytic property was investigated under the above optimal oligomerization conditions (Figure 9a). It should be noted that the activity of the three triazine COFs is proportional to the size of their specific surface area. Ni@MAPA-COF based on MAPA-COF with a para-substituent had the highest catalytic activity of 10.55 × 10^4^ g/(mol Ni·h) among the three nickel(II) catalysts for ethylene oligomerization (Figure 9a). This was because Ni@MAPA-COF had the highest specific surface area, pore volume (Figure 9b) and nickel content, which facilitates the access of the co-catalysts into the pores and the formation of more active centers. It also promotes the insertion of ethylene molecules and increases the rate of chain growth. Interestingly, Ni@MA*m*PA-COF, with the maximum pore width and higher nickel content (Table 1 and Table 2), had the lowest catalytic activity and highest selectivity of C_8+_ olefins in ethylene oligomerization (Figure 9a). The possible reason was that the lower specific surface area reduces the chances of contact between the active site and ethylene molecules, which leads to lower catalytic activity. The wide pore size is conducive to the chain growth of ethylene to produce more C_8+_ olefins. The catalyst Ni@MA*o*PA-COF with OPA as the guest building unit showed the best selectivity for C_4_, reaching 93.99% (Figure 9a). It is speculated that the effects of β-H elimination and chain transfer effect led to a various of oligomerization products (Figure 9a) [36,37]. However, due to the smallest pore width, Ni@MA*o*PA-COF cannot provide space conducive to the synthesis of long-chain olefins. Therefore, the ethylene molecule tends to undergo a β-H elimination reaction after growing to C_4_, which leads to the highest C_4_ yield being in the product catalyzed by Ni@MA*o*PA-COF. Conversely, Ni@MAPA-COF has the largest pore size, so the final products were composed of olefins with different carbon numbers due to the repeated chain extension and β-H elimination reactions, including C_6_ and C_8_.

### 2.3. The Reusability of Ni@MAoPA-COF

**Figure 10:**
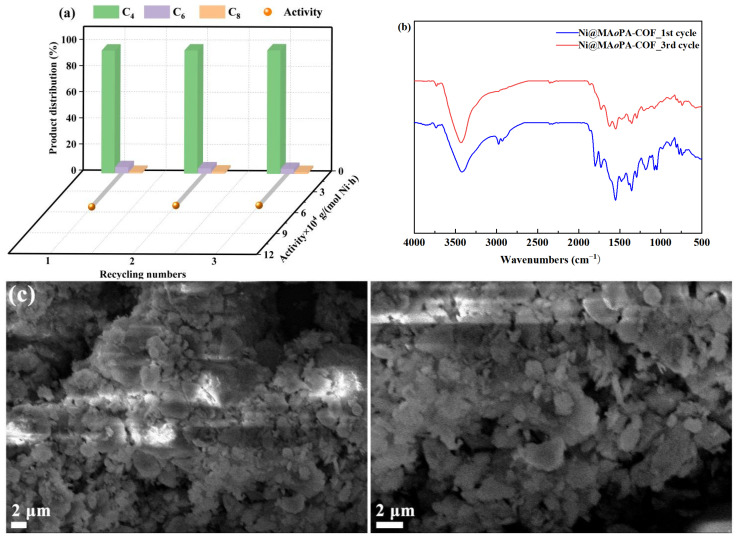
Recyclability of Ni@MA*o*PA-COF (**a**); FT-IR images of Ni@MA*o*PA-COF after the second and third cycles of ethylene oligomerization (**b**); SEM image of Ni@MA*o*PA-COF after the third cycle of ethylene oligomerization (**c**); reaction conditions: 2 µmol precatalyst, MAO (Al/Ni = 500), 20 mL cyclohexane, 25 °C, 0.5 MPa, 30 min.

The effect of the chemical structure on the catalytic performance showed that Ni@MAoPA-COF had a higher catalytic activity than Ni@MAmPA-COF in ethylene oligomerization, and Ni@MAoPA-COF has a larger specific surface area. In order to evaluate the reusability of triazine nickel(II) catalysts, the reusability of Ni@MA*o*PA-COF was investigated under the optimal oligomerization parameters due to its larger specific surface area and higher activity in ethylene oligomerization. After the initial oligomerization of ethylene, the reacted catalyst was collected by centrifugation, sonication and washing, and the obtained results are shown in Figure 10a. There was no significant decrease in catalytic activity as the reaction was repeated, and the selectivity value of C_4_ was consistently above 90% (Figure 10a). Compared with the IR spectrum of the fresh Ni@MAoPA-COF, slight changes in the IR spectra were observed after the third cycle and the characteristic peaks of the functional groups (-CHO, C=N and C-N) weakened (Figure 10b). This was possible because of the adsorption of residual oligomers on the catalyst surface and the residue of the deactivated cocatalyst, which further indicated the good stability of the synthesized Ni@MA*o*PA-COF. The SEM images showed that the catalyst presented as a blocky stacking pattern after the cycling experiment, which was smaller and more tightly packed than the fresh catalyst (Figure 10c). This is considered to be caused by the adsorption of residual oligomers on the catalyst surface. The adsorbed oligomers mask the active sites on the catalyst surface, and also lead to a subtle decrease in catalytic activity.

## 3. Experimental Methods

### 3.1. Materials and Characterization

Melamine, (DME)NiCl_2_, meta-phthalaldehyde (MPA) and ortho-phthalaldehyde (OPA) were purchased from Aladdin Chemistry Co., Ltd. (Shanghai, China). The 1,4-dioxane, anhydrous ethanol (EtOH), acetic acid (HAc) and DMSO used in the experiment were supplied by Tianjin Kermel Chemical Reagent Co., Ltd. (Tianjin, China). Methylaluminoxane (MAO), toluene and cyclohexane were purchased from Sigma-Aldrich (Shanghai, China). All solvents used in ethylene oligomerization were deoxygenated using desiccants and distilled prior to use.

Fourier transform infrared (FT-IR) spectroscopy was performed using a 6700 spectrometer (Nicolet Instrument Corp., Madison, WI, USA) with KBr pellets in the range of 4000–500 cm^−1^. Scanning electron microscopy (SEM) images were obtained with a Zeiss SIGMA (Jena, Germany) apparatus scanning electron microscope. Energy-dispersive X-ray spectrometry (EDX) with a silicon-drift detector was employed to investigate the elemental composition of the sample. Inductively coupled plasma atomic emission spectroscopy (ICP-AES) was performed to analyze the content of metal nickel. X-ray diffraction (XRD) patterns were obtained using the D/MAX-2200 X-ray diffractometer from Rigaku Co., Ltd. (Tokyo, Japan). Brunauer–Emmett–Teller (BET) measurements were carried out to calculate the specific surface areas on a TriStar II 3020 2.00 (Micromeritics Co., Ltd., Shanghai, China). XPS measurement was carried out on a Kalpha Thermo Fisher Scientific spectrometer equipped with a monochromatic Al Kα X-ray source. Thermogravimetric analysis (TGA) was performed using a Diamond TG/DTA Perkin-Elmer SII equipment (Perkin-Elmer, Waltham, MA, USA) from 30 to 900 °C at a heating rate of 10 °C/min under a nitrogen atmosphere. 

### 3.2. Synthesis of COFs Supports

Melamine (0.252 g, 2.00 mmol) and MPA (0.402 g, 3 mmol) were dissolved in 5 mL DMSO and 5 mL 1,4-dioxane under nitrogen atmosphere, respectively. The MPA solution was added into the melamine solution under a nitrogen atmosphere and 0.5 mL HAc (6 mol/L) solution was added to the mixture system. The mixture was heated at 120 °C for 72 h under nitrogen. After cooling down to room temperature, the mixture was filtrated to obtain the solid. And the solid was washed three times with DMSO, 1,4-dioxane and EtOH orderly. The final solid was vacuum-dried at 65 °C for 6 h to yield an orange–yellow solid (MA*m*PA-COF). The yield of MA*m*PA-COF was about 54%. IR (KBr, cm^−1^): 3394, 2920, 1561, 1494, 1340, 816.

The MA*o*PA-COF was synthesized in accordance with the similar procedure of MA*m*PA-COF using melamine (0.252 g, 2.00 mmol) and OPA (0.402 g, 3 mmol) in mixed solvent of DMSO and 1,4-dioxane with 6 mol/L HAc as catalyst. The yield of MA*o*PA-COF was about 37%. IR (KBr, cm^−1^): 3370, 2922, 1730, 1550, 1349, 807, 738.

### 3.3. Synthesis of COFs-Supported Nickel Catalysts

MA*m*PA-COF (0.136 g, 0.10 mmol) was dispersed in 10 mL EtOH. (DME)NiCl_2_ (0.132 g, 0.60 mmol) was dissolved in 10 mL EtOH and the solution was added dropwise to the MA*m*PA-COF solution. The mixture was stirred and refluxed at 80 °C for 12 h under nitrogen. After cooling down to room temperature, the product obtained by filtration was washed with EtOH more than three times to remove the unreacted (DME)NiCl_2._ The product was finally dried under vacuum at 100 °C for 6 h to yield a pale yellow solid (Ni@MA*m*PA-COF). The yield of Ni@MA*m*PA-COF was about 45%. IR (KBr, cm^−1^): 3394, 2920, 1561, 1494, 1340, 816. Anal. by EDX (atomic %) for [C_33_H_18_N_15_(NiCl_2_)_3_]_n_ (1013n): C 44.48, N 45.95, O 8.44, Ni 1.13. Anal. by ICP (atomic %): Ni 5.76.

Ni@MA*o*PA-COF (orange–yellow solid) was synthesized in accordance with the similar procedure of Ni@MA*m*PA-COF using MA*o*PA-COF (0.136 g, 0.10 mmol) and (DME)NiCl_2_ (0.132 g, 0.60 mmol) as materials, and the yield of Ni@MA*o*PA-COF was about 56%. IR (KBr, cm^−1^): 3370, 2922, 1730, 1550, 1349, 807, 738. Anal. by EDX (atomic %) for [C_22_H_12_N_10_(NiCl_2_)_2_]_n_ (546n): C 58.93, N 30.32, O 9.95, Ni 0.80. Anal. by ICP (atomic %): Ni 4.91.

### 3.4. Catalytic Performance of Catalyst

Catalytic oligomerization was conducted in a 250 mL stainless steel autoclave with a magnetic stirrer inside and equipped with an electronic pressure sensor. Before the experiment, the reactor was evacuated and flushed with N_2_ and ethylene three times to remove residual moisture. The pre-catalyst was suspended in cyclohexane in the Schlenk flask and the resulting solution was immediately injected into the autoclave under nitrogen. Then, the necessary amount of cocatalyst solution dissolved in additional toluene was loaded into the reactor. The reactor was flushed with ethylene, and ethylene was continuously fed to the autoclave to provide constant pressure. After the catalytic experiment, the reactor was cooled to 0 °C under stirring, and then excess ethylene was vented out. The reaction was quenched by a solution of HCl in ethanol (10 vol%). The organic phase was further separated from the pre-catalyst and the organic compounds were characterized with GC to determine the composition and product distribution.

### 3.5. Recycling Experiment

In order to assess the stability of the synthesized catalysts, recycling tests were performed. When the oligomerization reaction was over, the reacted mixture was washed several times with the dilute HCl (10%) solution of ethanol under nitrogen atmosphere using the standard Schlenk technique to remove MAO from the reaction. Then, the upper clear liquid was withdrawn using a syringe at every turn, then the spent catalyst was collected using centrifugation. The catalyst was further washed several times in ethanol and cyclohexane to remove impurities. Finally, the regenerated catalyst was vacuum-dried at 200 °C for 6 h. The reaction was carried out again as described above for ethylene oligomerization, and the same procedure was repeated in the subsequent recycling.

## 4. Conclusions

In this study, ethylene oligomerization was performed using triazine nickel(II) catalysts with aldehyde groups in the meta- and ortho-positions. The morphology, coordination mode, specific surface area and pore width of the two nickel(II) catalysts are affected by the substituent positions of the sub-constructing units. This also affects their catalytic performance in ethylene oligomerization. The Ni@MA*o*PA-COF catalyst produced high catalytic activity and high selectivity for C_4_ oligomers. This indicates that catalysts with a large specific surface area are more favorable for reactant contacting, but the smaller pore width limits the chain growth reaction of the ethylene molecule, allowing the oligomeric products to concentrate on butene. Moreover, Ni@MA*o*PA-COF showed good stability during recycling, and the selectivity of butene, which was consistently above 90%, in catalyst recycling was repeated.

## Figures and Tables

**Table 1:** The content of each element in three nickel(II) catalysts measured by EDX and ICP-AES.

Samples	EDX				ICP
	C	N	O	Ni	Ni
Ni@MAPA-COF	81.35	8.94	8.38	1.33	7.58
Ni@MA*m*PA-COF	44.48	45.95	8.44	1.13	5.76
Ni@MA*o*PA-COF	58.93	30.32	9.95	0.80	4.91

**Table 2:** Textural properties for COFs and COFs-based catalysts.

Sample Name	BET Surface Area (m^2^ g^−1^)	Pore Width (nm)	Pore Volume (cm^3^ g^−1^)
MA*m*PA-COF ^a^	32.8	10.0	0.06
MA*o*PA-COF ^a^	64.9	13.7	0.23
MAPA-COF ^b^	234.7	17.5	0.33
Ni@MA*m*PA-COF	16.3	18.3	0.08
Ni@MA*o*PA-COF	40.3	1.9	0.06
Ni@MAPA-COF	192.6	17.0	0.30

^a^ Condition: mixed solvent (DMSO:1, 4-dioxane = 1:1), temperature = 120 °C, time = 72 h; ^b^ condition: single solvent (DMSO), temperature = 150 °C, time = 72 h.

**Table 3:** The gray comprehensive correlation degree of each factor and different target.

Target	Molar Ratio of Al/Ni	Reaction Time	Reaction Pressure
Activity	0.6764	0.5776	0.7238
Selectivity of C_4_ + C_6_	0.6824	0.5818	0.6536

## Data Availability

The original contributions presented in this study are included in the article. Further inquiries can be directed to the corresponding authors.
